# Callitrichine herpesvirus 3 in the common marmoset is a model of Epstein-Barr virus infection and associated lymphoma

**DOI:** 10.1371/journal.ppat.1014450

**Published:** 2026-07-17

**Authors:** Stacey L. Piotrowski, Xiaofan Li, Caitlin E. Fitzpatrick, Allison Tucker, Amanda Lee, Emily Leibovitch, Maria Chiara G. Monaco, Anna K. Grosskopf, Rose Peterson, Jennifer E. Dwyer, Andrew Warner, Matthew F. Starost, R. Mark Simpson, Stacey J. Sukoff Rizzo, Afonso C. Silva, Krystal Allen-Worthington, Heather Narver, Laurie T. Krug, Steven Jacobson

**Affiliations:** 1 Viral Immunology Section, National Institute of Neurological Disorders and Stroke (NINDS), National Institutes of Health (NIH), Bethesda, Maryland, United States of America; 2 HIV and AIDS Malignancy Branch, National Cancer Institute (NCI), NIH, Bethesda, Maryland, United States of America; 3 Laboratory of Cancer Biology and Genetics, Center for Cancer Research, NCI, NIH, Bethesda, Maryland, United States of America; 4 Translational Neuroradiology Section, NINDS, NIH, Bethesda, Maryland, United States of America; 5 Bioinformatics Core, NINDS, NIH, Bethesda, Maryland, United States of America; 6 Molecular Histopathology Laboratory, NCI, Frederick National Laboratory for Cancer Research, Frederick, Maryland, United States of America; 7 Division of Veterinary Resources, NIH, Bethesda, Maryland, United States of America; 8 Department of Neurobiology and Aging Institute, University of Pittsburgh School of Medicine, Pittsburgh, Pennsylvania, United States of America; 9 Veterinary Medicine and Resources Branch, National Institute of Mental Health, NIH, Bethesda, Maryland, United States of America; 10 Animal Health and Care Section, NINDS, NIH, Bethesda, Maryland, United States of America; University of Wisconsin-Madison, UNITED STATES OF AMERICA

## Abstract

Herpesviruses, such as Epstein-Barr virus (EBV), are thought to potentially play a significant role in multiple disease processes, including neoplasia, multiple sclerosis (MS), and more recently, Alzheimer’s disease (AD). Animal models remain vital tools for understanding these diseases and developing therapeutics. Callitrichine herpesvirus 3 (CalHV-3) was identified in the early 2000s in the common marmoset (*Callithrix jacchus*). Although phylogenetically related to human EBV, the biological similarities between CalHV-3 and EBV have not been thoroughly characterized. Over 450 marmosets from five biomedical research colonies in the United States were screened for CalHV-3 using droplet digital PCR (ddPCR). Peripheral blood mononuclear cells (PBMCs) were magnetically separated to determine viral loads in B-cell enriched and B-cell depleted populations. A CalHV-3 infected cell line was reactivated to determine gene expression profiles using quantitative-Reverse Transcription PCR (q-RT-PCR). Archived cases of lymphoma in the marmoset were immunophenotyped by immunohistochemistry (IHC). In the neoplastic tissue, CalHV-3 viral loads were measured by ddPCR, and viral transcripts were visualized using RNAscope. The prevalence of CalHV-3 in these research colonies ranged from 19-63%. The virus was detected longitudinally in PBMCs and saliva. Infected marmosets had CalHV-3 viral loads enriched in B-cells. All cases of B-cell lymphoma in the marmoset were positive for CalHV-3 DNA, with transcripts of EBV latent and lytic gene homologs detected in neoplastic tissue. Like EBV, CalHV-3 is characterized by persistent infection, shedding in saliva, B-cell tropism, latent and lytic gene expression profiles, and lymphomagenesis in a subset of infected animals. These results further suggest that CalHV-3 in the common marmoset may serve as a translational model of EBV infection and associated diseases.

## Author summary

Over 90% of the worldwide population will be infected with Epstein-Barr virus (EBV) during their lifetime. In addition to causing mononucleosis, EBV has also been associated with a growing number of diseases, including cancer, multiple sclerosis, and Alzheimer’s disease. Despite these associations, treatments specific for EBV are currently limited. Studying closely related viruses in other species, such as nonhuman primates, remain important tools to further our understanding of EBV and its role in disease. In this work, we further characterize the similarities between EBV and a virus that infects the common marmoset, Callitrichine herpesvirus 3 (CalHV-3). We found that CalHV-3 shares many important similarities with EBV, including the ability to cause cancer in a small subset of infected animals. Our study provides important validation of an animal model that can be utilized in future studies to better understand EBV biology and disease processes, which may ultimately lead to new EBV-focused therapeutics and preventatives.

## Introduction

Epstein-Barr virus (EBV), also known as human herpesvirus 4, is a ubiquitous gammaherpesvirus that persists in most affected individuals as a lifelong, asymptomatic infection [1,2]. In addition to being the primary cause of infectious mononucleosis, EBV has also been associated with a variety of cancers, lymphoproliferative diseases, and more recently, neurodegenerative disorders such as multiple sclerosis (MS) and Alzheimer’s disease [3–5]. Despite ongoing research studies and clinical trials, preventatives and therapeutics for EBV remain limited [6,7].

Animal models are essential research tools that aid in the understanding of human viral infections and diseases [6,8]. In general, herpesviruses like EBV are species-specific, limited by the tropism to their natural host, which makes experimental infection of animals with human herpesviruses challenging [9,10]. However, the study of animal herpesviruses that are natural pathogens in other species, such as nonhuman primates (NHPs) which have the closest phylogenetic relationship to humans, can be utilized as EBV infection and disease models [9,11]. The conservation of biologic and pathogenic properties of orthologous gammaherpesviruses in NHP species likely more closely mimics human EBV infection than other animal models, such as rodents, whose endogenous herpesviruses may lack properties of EBV, such as efficient B-cell transformation in culture and homologs of EBV genes with oncogenic properties [12].

In the early 2000s, a lymphoproliferative disease was noted in a biomedical research colony of common marmosets (*Callithrix jacchus)*, a New World NHP, and associated with a newly identified herpesvirus [13]. Named Callitrichine herpesvirus 3 (CalHV-3), genomic sequencing of the virus confirmed its classification as an EBV-related lymphocryptovirus with positional homologues to EBV lytic and latent genes [14–16] (S1 Fig). More recently, studies related to the possible role of gammaherpesviruses in MS have utilized CalHV-3 in the marmoset as a model system [17–21]. While CalHV-3 infection has been documented in both research colonies and the native Brazilian population of marmosets, the biological properties of the virus have not been thoroughly described [22,23].

The goal of this study was to characterize the biological similarities of CalHV-3 infection in the common marmoset to human EBV, further exploring its utility as a translational experimental model of EBV infection and associated diseases. PBMC samples from over 450 total marmosets from five research colonies were screened for CalHV-3, with colony prevalence ranging from 19-63%. In a subset of animals, longitudinal PBMC and saliva samples showed persistent infection and viral load fluctuations, particularly in saliva. CalHV-3 viral load was enriched in B-cells, confirming B-cell tropism. While most infections were clinically asymptomatic, of significant interest was the observation that in cases of marmoset B-cell lymphoma, all (9/9, 100%) were positive for CalHV-3 with extremely high viral DNA loads (tens of millions of copies) and viral gene expression in neoplastic tissue, suggesting that CalHV-3 is an oncogenic virus and is the cause of B-cell lymphoma in the marmoset. These biological similarities to human EBV suggest that CalHV-3 in the common marmoset can be utilized as a translational research model of gammaherpesvirus infection.

## Results

### Prevalence of CalHV-3 in marmoset colonies by ddPCR

**Fig 1:**
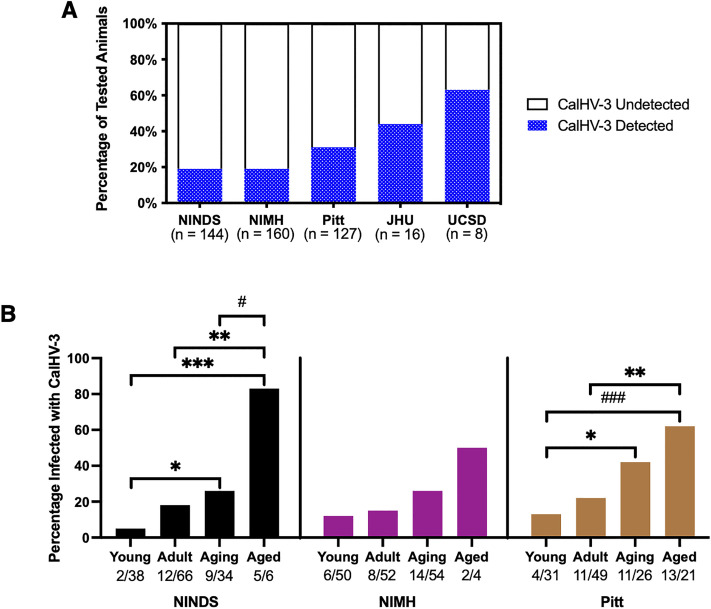
CalHV-3 was detected in all tested colonies. **(A)** PBMCs from over 450 marmosets from five colonies were screened for CalHV-3 via ddPCR. CalHV-3 was detected in all colonies. Prevalence of CalHV-3 in tested colonies ranged from 19-63%. **(B)** In the NINDS and University of Pittsburgh (Pitt) colonies, there was a significantly higher prevalence of CalHV-3 infection in aged animals when compared to adult and young animals. There was also a significantly higher prevalence in aging animals compared to young animals in both colonies. In the NINDS colony, aged animals also had a significantly higher prevalence of infection compared to aging animals. There were no statistically significant differences in CalHV-3 prevalence between any age groups in the NIMH colony. #p = 0.01, *p = 0.02, **p = 0.002; ***p = 0.0001, ###p = 0.0006; Fisher’s exact test.

To characterize the prevalence of CalHV-3 in common marmoset colonies used in biomedical research, large-scale screening was implemented in the NINDS (October-December 2021), the NIMH (September 2022), and the University of Pittsburgh MARMO-AD colonies (July-December 2022). PBMCs were isolated from whole blood samples collected during approved routine health surveillance or experimental procedures. DNA was extracted from PBMCs for ddPCR that targeted both the CalHV-3 *gp07* viral polymerase and the marmoset housekeeping gene *beta-actin*. The prevalences of CalHV-3 in the NINDS, NIMH, and the University of Pittsburgh colonies (Pitt) were 19% (28/144), 19% (30/160), and 31% (39/127), respectively (Fig 1A).

Smaller importations of marmosets from outside colonies were also screened for CalHV-3 during the quarantine period prior to introduction to the NIMH colony. CalHV-3 was also detected in imported animals from Johns Hopkins University (JHU; October 2023) (7/16, 44%) and the University of California at San Diego (UCSD; December 2023) (5/8, 63%) (Fig 1A).

### Prevalence of CalHV-3 infection was significantly higher in aged marmosets

In general, in the three colonies with large-scale screening (NINDS, NIMH, University of Pittsburgh), the prevalence of CalHV-3 infection increased with age (Fig 1B). In both the NINDS and University of Pittsburgh colonies, a significantly higher prevalence of CalHV-3 infection was associated with aged animals (>60%) when compared to adult (18–22%) and young animals (<15%) (**p = 0.002; ***p = 0.0001, ###p = 0.0006; Fisher’s exact test). There was also a significantly higher prevalence in aging animals (26–42%) compared to young animals (<15%) in both colonies (*p = 0.02, Fisher’s exact test). Additionally, in the NINDS colony, aged animals (83%) also had a significantly higher prevalence of infection when compared to aging animals (26%) (#p = 0.01, Fisher’s exact test). There were no statistically significant differences in prevalence between any age groups in the NIMH colony, likely due to the comparatively small sample size of aged animals (n = 4) (p > 0.05, Fisher’s exact test).

All (16/16, 100%) animals screened from the JHU cohort were adults, and 88% (7/8) of the UCSD marmosets were young, with the remaining animal being an adult. Therefore, age prevalence analyses were not performed on the JHU and UCSD colonies. All tested colonies had an equal distribution of each sex in CalHV-3 infected animals (S2 Fig). There was no association between sex and CalHV-3 status in any colony (p > 0.05, Fisher’s exact test). In summary, the accrual pattern with age supports a link between exposure and a cumulative incidence of natural transmission in marmosets housed in colonies across institutions.

### Mean CalHV-3 viral loads did not significantly differ between colonies, age groups, or sex

**Fig 2:**
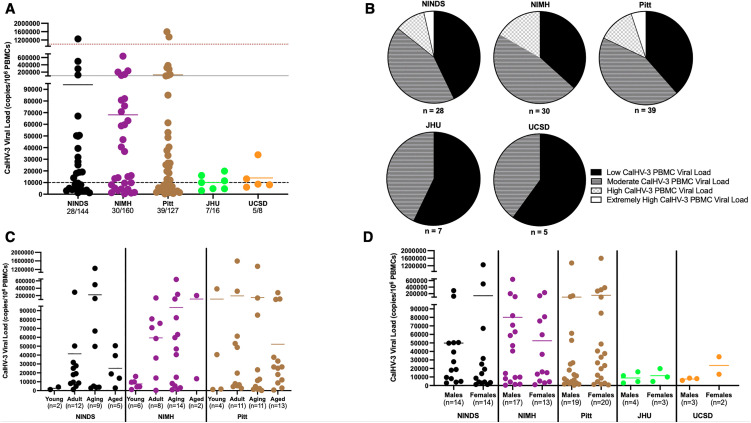
Mean CalHV-3 viral loads did not differ between colonies, age groups, or sexes. **(A)** There was no statistically significant difference in the mean positive CalHV-3 viral load in PBMCs between colonies (p > 0.05, one-way ANOVA). CalHV-3 PBMC viral loads included low (below the black dashed line), moderate (between the black dashed line and gray solid line), high (between the gray solid line and dotted red line), and extremely high (above the dotted red line). **(B)** Most marmosets positive for CalHV-3 had low or moderate PBMC viral loads in all tested colonies. A smaller percentage of animals had high viral loads in the NINDS, NIMH, and Pitt colonies, with rare animals having extremely high viral loads in the NINDS and Pitt colonies. **(C)** There was no statistically significant difference in the mean positive CalHV-3 viral load between age groups within the same colony (p > 0.05, one-way ANOVA). **(D)** Within each colony, there was no statistically significant difference in the mean positive CalHV-3 viral load between sexes (p > 0.05, unpaired t-test). Data are represented as the mean positive CalHV-3 viral load.

The mean viral load of CalHV-3-positive animals was determined in all tested colonies (Fig 2A). Mean viral loads ranged from 9,987 copies/10^6^ PBMCs in the JHU cohort to 125,529 copies/10^6^ PBMCs in the University of Pittsburgh colony. However, there was no statistically significant difference in the mean positive CalHV-3 viral load in PBMCs between colonies (p > 0.05, one-way ANOVA).

CalHV-3 PBMC viral loads were arbitrarily subdivided into the following categories: low (less than 10,000 copies of CalHV-3/10^6^ PBMCs), moderate (10,000–100,000 copies of CalHV-3/10^6^ PBMCs), high (100,001–1,000,000 copies/10^6^ PBMCs), and extremely high (more than 1,000,000 copies/10^6^ PBMCs) (Fig 2A). Most marmosets positive for CalHV-3 on ddPCR had low (37–60%) or moderate (40–47%) PBMC viral loads in all tested colonies (Fig 2B). A smaller percentage of animals (11–17%) had high viral loads in the NINDS, NIMH, and University of Pittsburgh colonies, with rare animals (4–5%) having extremely high viral loads in the NINDS and University of Pittsburgh colonies (Fig 2B).

The mean CalHV-3 viral load for CalHV-3 positive animals was calculated for each age group in the NINDS, NIMH, and University of Pittsburgh colonies (Fig 2C). There was no statistically significant difference in the mean CalHV-3 viral load between age groups within the same colony (p > 0.05, one-way ANOVA). The mean viral loads in age groups were not calculated in the JHU and UCSD animals due to the low numbers of animals in age groups in these tested populations. Mean viral loads for CalHV-3-positive males and females were calculated for each colony (Fig 2D). Within each colony, there was no statistically significant difference in the mean CalHV-3 viral load between sexes (p > 0.05, unpaired t-test).

### CalHV-3 was detectable in the saliva of most infected animals

A total of 96 saliva samples, along with a corresponding whole blood sample, were collected (June 2022-October 2023) from a subset of 49 NINDS marmosets to determine if CalHV-3 was shed in the saliva of infected animals and if CalHV-3 saliva viral loads correlated with PBMC viral loads. Twenty marmosets, including both CalHV-3 infected and uninfected animals, had contemporaneous saliva and PBMC samples collected at multiple different timepoints, ranging from 2-8 timepoints per animal.

**Fig 3:**
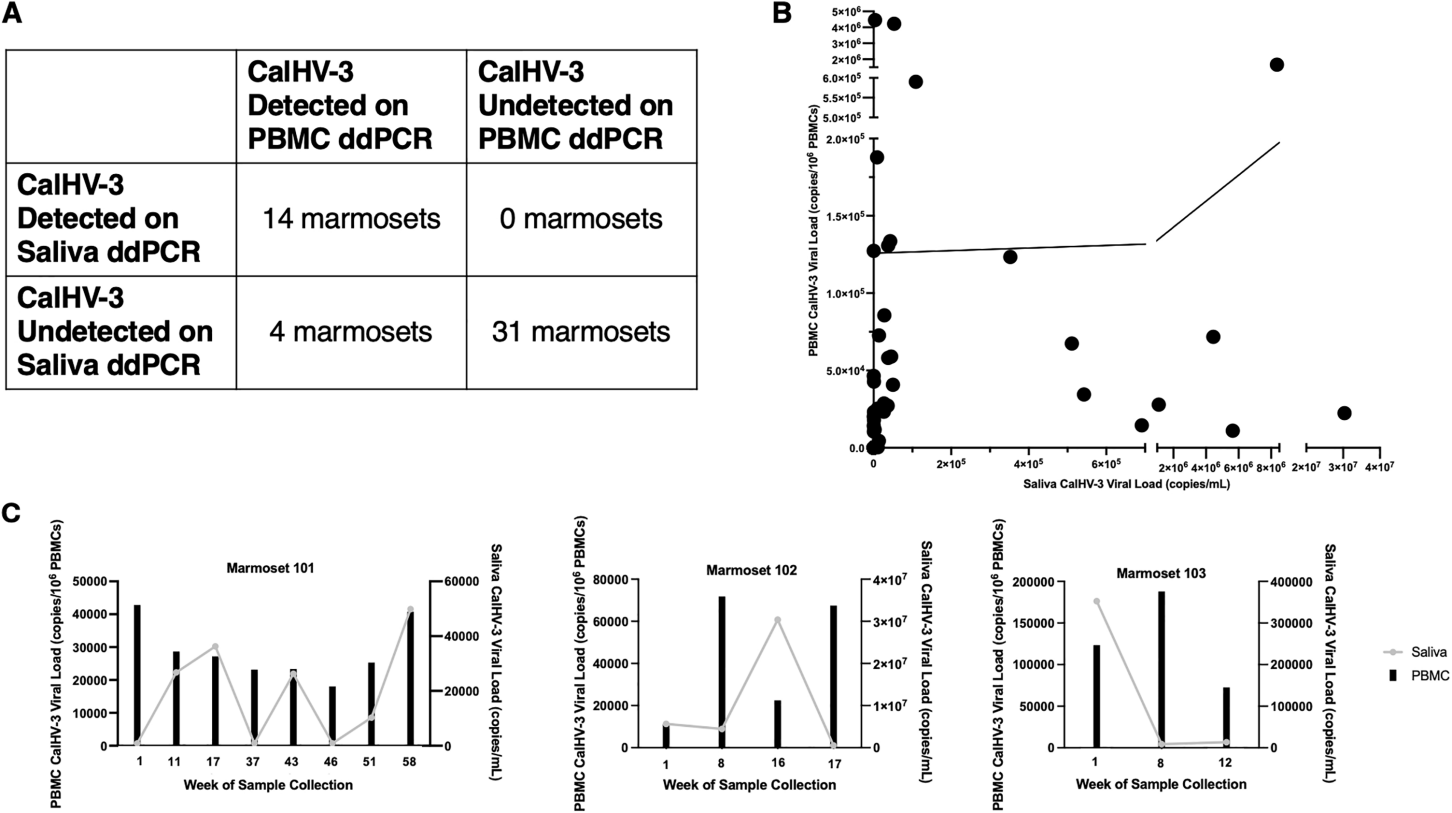
CalHV-3 was detected in both PBMCs and saliva, with large fluctuations in salivary viral loads. **(A)** Detection of CalHV-3 in PBMCs was significantly associated with detection of CalHV-3 in saliva (p < 0.0001, Fisher’s exact test). **(B)** There was no evidence of a correlation between the saliva viral load and PBMC viral load in contemporaneous samples (r = 0.04, p = 0.68). **(C)** In three CalHV-3 positive animals with at least three sample collection timepoints, virus was detected in saliva (grey lines) and PBMCs (black bars) longitudinally. Viral loads fluctuated over time in infected animals, particularly in saliva which showed up to ten-fold changes in viral load in the same animal.

From the 49 animals that had saliva collected, 18 animals were positive for CalHV-3 in PBMCs, and 31 were CalHV-3 negative in PBMCs (S3 Fig). Detection of CalHV-3 in PBMCs was significantly associated with the detection of CalHV-3 in saliva (p < 0.0001, Fisher’s exact test) (Fig 3A). All (31/31, 100%) animals negative for CalHV-3 in PBMCs were also negative in saliva (Figs 3A and S3). By contrast, of the 18 animals that were CalHV-3 positive in PBMCs, 14 (78%) were also positive in saliva (Figs 3A and S3). 22% (4/18) of CalHV-3 infected animals positive in PBMCs did not have CalHV-3 detected in saliva (Figs 3A and S3). Of these four animals, two had a second saliva sample collected at a different timepoint (S3 Fig). In one of these animals, the second saliva sample approximately two months later was positive for CalHV-3, while CalHV-3 remained undetectable in the saliva of the other marmoset (S3 Fig). The sensitivity and specificity of saliva CalHV-3 detection compared to PBMCs were 78% and 100%, respectively, with a positive predictive value of 100% and a negative predictive value of 89%.

To determine if there was a correlation between viral loads in PBMCs and saliva, corresponding saliva (copies/mL) and PBMC (copies/10^6^ PBMCs) viral loads were plotted for the 96 total samples from the 49 animals, and the Pearson r correlation coefficient was calculated. There was no evidence of a correlation between the saliva viral load and PBMC viral load (r = 0.04, p = 0.68) (Fig 3B).

### CalHV-3 remained detectable in infected animals over time, with large fluctuations in saliva viral loads

Ten marmosets had three or more longitudinal saliva and PBMC samples, including seven animals that were undetected for CalHV-3 at all collected timepoints. In the three CalHV-3-positive animals with samples collected at three or more time points, the virus was detected in saliva and PBMCs at all collected timepoints. Viral loads in PBMCs remained relatively consistent over time, while salivary viral loads fluctuated in CalHV-3 infected animals, with greater than ten-fold increases in salivary viral loads in the same animal at different sample collection timepoints in some cases (Fig 3C).

### CalHV-3 was enriched in marmoset B-cells

To determine whether CalHV-3 is B-cell tropic similar to EBV, PBMCs from ten animals from the NINDS and NIMH colonies were separated into B-cell enriched and B-cell depleted populations. Based on sample volume and number of PBMCs recovered, flow cytometry was performed on a subset of these ten animals to characterize the cellular composition of the separated populations (S4 Fig and Animals G-J in Table 1). In the four PBMC samples analyzed by flow cytometry, the percentage of CD20 positive B-cells ranged from 9-39%, while the positively selected B-cell enriched population ranged from 72-89% CD20 positive B-cells (S4 Fig).

**Table 1:** CalHV-3 viral loads were enriched in B-cells compared to PBMCs and B-cell depleted populations.

Animal	PBMC CalHV-3 Viral Load (copies of CalHV-3 /10^6^ PBMCs)	B-cell Depleted Population CalHV-3 Viral Load (copies of CalHV-3 /10^6^ cells)	B-cell Enriched Population CalHV-3 Viral Load (copies of CalHV-3 /10^6^ cells)
A	945,197	177,560	4,285,714
B	262	99	10,602
C	undetected	undetected	undetected
D	204,100	3,511	948,799
E	undetected	undetected	undetected
F	194,373	9,748	1,042,182
G	17,127	982	154,664
H	undetected	undetected	undetected
I	14,534	5,102	244,703
J	undetected	undetected	undetected

CalHV-3 viral loads were measured by ddPCR in the PBMCs, the negatively selected B-cell depleted population, and the positively selected B-cell enriched population for each sample (Table 1). CalHV-3 was not detected in the PBMCs of four animals, and it remained undetected in the magnetically separated B-cell enriched and B-cell depleted populations (Table 1; Animals C, E, H, J). Six animals were positive for CalHV-3 in PBMCs, with a range of viral loads (Table 1; Animals A-B, D, F-G, I). When viral loads were compared between the three cell populations in the same animal, viral loads were decreased in the B-cell depleted population compared to PBMCs. Importantly, CalHV-3 was enriched in the B-cell population, with increased viral loads compared to PBMCs. For example, in Animal G (Table 1), the CalHV-3 viral load in the B-cell enriched population was nine times higher than that observed in PBMCs.

### CalHV-3 was associated with all cases of B-cell origin lymphoma in the common marmoset

During the initial large-scale screening of colonies, one animal in the NINDS colony (1/144, 0.7%) was identified with an extremely high viral load of over a million copies of CalHV-3 per million PBMCs (Fig 2A). This animal was subsequently euthanized due to being unresponsive to treatment for chronic weight loss and diarrhea. Histopathology findings were suggestive of lymphoma (Case 7). Based on this case and the original identification of CalHV-3 in relation to lymphoproliferative disease [13,14], the association between CalHV-3 and lymphoma in the common marmoset was further investigated.

**Fig 4:**
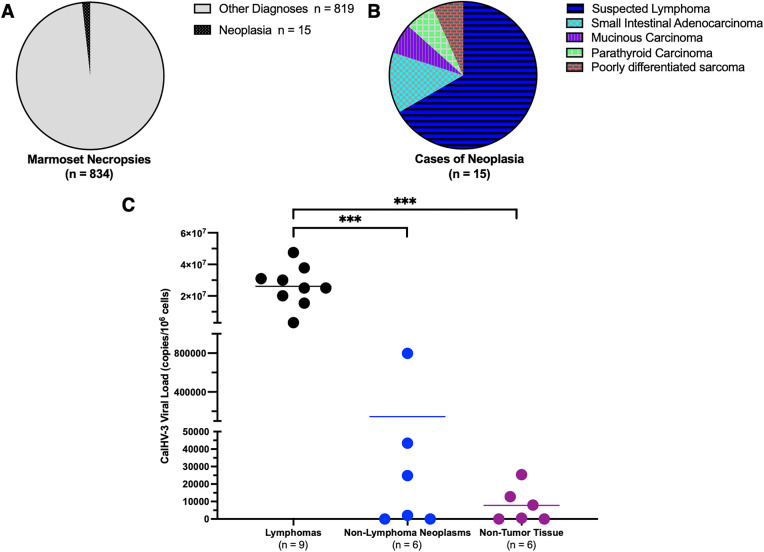
CalHV-3 was associated with all cases of B-cell lymphoma in the common marmoset. **(A)** Of the 834 marmoset necropsies performed during the thirty-year query of the NIH DVR archives, 15 (1.8%) were diagnosed with neoplasia postmortem. **(B)** Based on H&E histopathology, lymphoma was suspected in 10 of the 15 cases of neoplasia (67%). **(C)** There was a statistically significant difference in the mean CalHV-3 viral load between B-cell lymphomas, non-lymphoma neoplasms, and non-tumor tissue. (p < 0.0001, one-way ANOVA). The mean viral load in lymphoma tissue (26.7x10^6^ copies of CalHV-3/10^6^ cells) was significantly higher than non-lymphoma neoplasms (1.4x10^5^ copies of CalHV-3/10^6^ cells) and patient matched nonneoplastic kidney (7.8x10^3^ copies of CalHV-3/10^6^ cells) (***p = 0.0003, unpaired t-tests). There was no statistically significant difference in the mean viral load between non-lymphoma neoplasms and non-tumor tissue (p > 0.05, paired t-test). Data are represented as means ± SD.

A search of the NIH DVR archives was performed for cases accessioned from 1992 through 2022 to identify marmosets diagnosed with neoplasia (S5 Fig). Of the 834 marmoset necropsies performed during these thirty years, 15 (1.8%) were diagnosed with neoplasia (Figs 4A and S5). Lymphoma was suspected in 10 (67%) cases based on H&E histopathology and morphology of a round cell tumor, including the case described above (Figs 4B and S5). Nine of those suspected lymphomas had tissue available for further analysis (S5 Fig, Cases 1–9). In 2023, another case of suspected lymphoma was identified postmortem in an animal with an extremely high viral load of over a million copies of CalHV-3 per million PBMCs, bringing the total number of suspected lymphoma cases with available tissue to ten (S5 Fig, Case 10). The five other cases of neoplasia included two small intestinal adenocarcinomas (13%), a mucinous carcinoma (7%), a parathyroid carcinoma (7%), and a poorly differentiated sarcoma (7%) (Cases 11–15) (S5 Fig and 6, Cases 11–15). Demographics and clinical and gross findings for all fifteen neoplasia cases analyzed in this study are presented in S2 Table.

**Fig 5:**
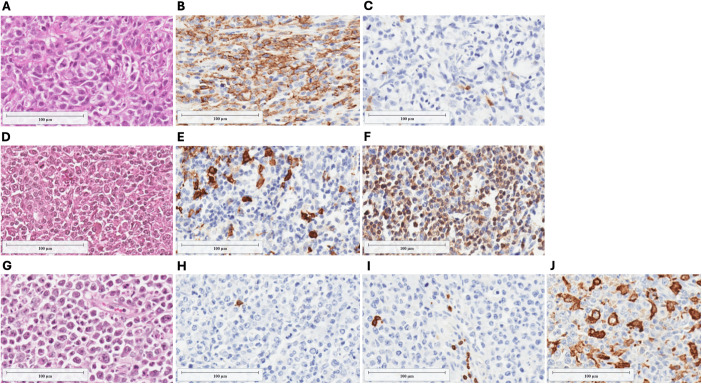
All lymphomas in the common marmoset were B-cell origin. IHC was performed to determine the neoplastic cell phenotype in cases of suspected lymphoma in the common marmoset. In Case 2, the neoplastic cells **(A)** are positive for CD20 **(B)** and negative for CD3 **(C)**, confirming a B-cell lymphoma. A total of seven cases were confirmed as B-cell lymphomas. A subtype of B-cell lymphoma, a T-cell-rich large B-cell lymphoma (TCRLBCL), was confirmed in two cases, characterized by a round cell neoplasm **(D)** consisting of a smaller population of atypical neoplastic CD20 positive B-cells **(E)** and a heavy infiltration of phenotypically normal CD3 positive T-cells **(F)** (Case 1). One suspected lymphoma (Case 9, **G**) was negative for CD20 **(H)** and CD3 **(I)** but positive for Iba1 **(J)**, consistent with a histiocytic sarcoma. **A**, **D**, **G**- H&E. **B**-**C**, **E**-**F**, **H**-**J**- DAB chromogen IHC.

H&E slides for the ten cases of suspected lymphoma and the five other neoplasms were reviewed, and initial diagnoses were confirmed. Anti-CD3 (T-cell marker) and anti-CD20 (B-cell marker) IHC were performed on these ten suspected lymphoma cases to immunophenotype the neoplasms. IHC of seven suspected lymphomas confirmed a B-cell lymphoma with CD20 positive and CD3 negative neoplastic cells (Fig 5A–5C) (Cases 2, 4–8, 10). In two other cases (Cases 1 and 3), a subtype of B-cell lymphoma, a T-cell-rich large B-cell lymphoma (TCRLBCL), was confirmed by a large population of morphologically normal CD3 positive T-cells and a smaller population of CD20 positive atypical neoplastic B-cells (Fig 5D–5F). One suspected lymphoma (Case 9) was negative for both CD3 and CD20 on IHC (Fig 5G–5I). Additional IHC for Iba1 (histiocytic marker) was performed on Case 9, confirming histiocytic sarcoma in this case (Fig 5J).

DNA was extracted from microtome sections of FFPE blocks of neoplastic tissue for the nine cases of confirmed B-cell origin lymphoma, the histiocytic sarcoma, and the five other non-lymphoma cell neoplasms. DNA was also extracted from non-neoplastic tissue (kidney) in the case of histiocytic sarcoma and the five cases of non-lymphoma neoplasms (Cases 9, 11–15). All nine cases of B-cell origin lymphoma, including the two instances of TCRLBCL, were positive by ddPCR for CalHV-3 DNA in the neoplastic tissue, with extremely high viral loads ranging from 3.1x10^6^-4.8x10^7^ copies of CalHV-3 per million cells (S3 Table and Fig 4C). Four of the other neoplasms (Cases 11–12, 14–15) were also positive for CalHV-3 in both neoplastic and non-neoplastic tissue but with comparatively lower viral loads in the low to moderate range (2.1x10^3^-8.0x10^5^ copies of CalHV-3/10^6^ cells). CalHV-3 was not detected in both the neoplastic and non-neoplastic tissue in the cases of histiocytic sarcoma and poorly differentiated sarcoma (Cases 9 and 13). One-way ANOVA analysis showed a statistically significant difference in the mean CalHV-3 viral load between B-cell lymphomas, non-lymphoma neoplasms, and non-tumor renal tissue from the non-lymphoma neoplasms (p < 0.0001) (Fig 4C). The mean viral load in lymphoma tissue (2.7 x10^7^ copies of CalHV-3/10^6^ cells) was significantly higher (>100-fold higher) than non-lymphoma neoplastic tissue (1.4x10^5^ copies of CalHV-3/10^6^ cells) and non-tumor tissue (7.8x10^3^ copies of CalHV-3/10^6^ cells) (***p = 0.0003, unpaired t-tests). There was no statistically significant difference in the mean viral load between non-lymphoma neoplasms and patient-matched renal tissue (p > 0.05, paired t-test).

### Expression of CalHV-3 homologs of EBV lytic genes was induced upon treatment of latent cells with reactivation stimuli

The CJ0149 cell line was derived from the primary culture of a marmoset B-cell lymphoma and harbors CalHV-3 [14]. The changes in viral gene expression upon treatment of CJ0149 cells to induce reactivation from latency have not been reported. Treatment of cells latently infected with the related human gammaherpesvirus EBV with tetradecanoyl phorbol acetate (TPA) induces the expression of the EBV lytic transactivator protein ZTA through activation of protein kinase C (PKC) to drive reactivation and genome-wide gene expression [24]. Sodium butyrate (NaB) is an inhibitor of histone deacetylases that leads to a broad increase in chromatin accessibility and gene expression [25]. For viral gene expression, CalHV-3 transcripts spanning genes homologous to EBV latency genes were expected to be constitutively expressed. In contrast, homologs of EBV lytic genes were expected to increase to much higher levels in a time-dependent manner.

**Fig 6:**
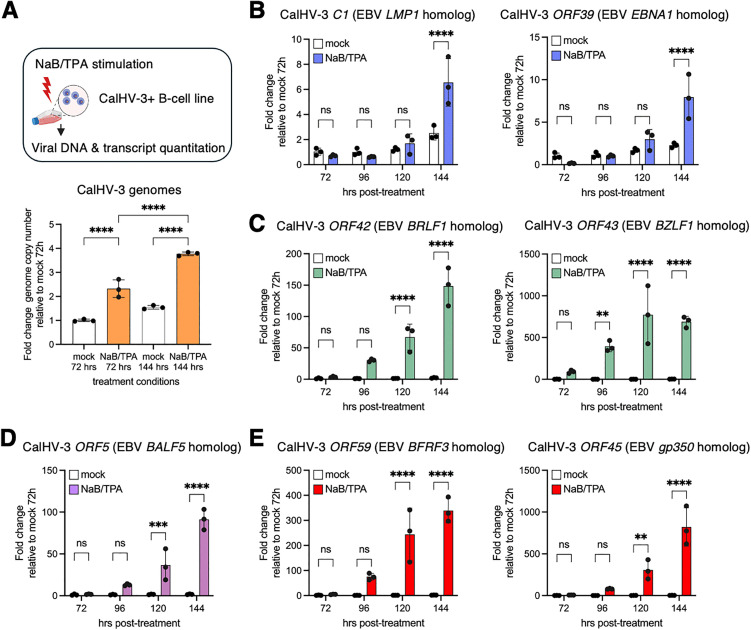
CalHV-3 gene expression was responsive to reactivation stimuli in the infected marmoset B-cell lymphoma cell line CJ0149. **(A)** CJ0149 cells were either mock treated or treated with 5 mM NaB and 20 ng/ml TPA to induce reactivation, as analyzed by measuring changes in viral DNA and viral transcripts. CalHV-3 viral genome copy numbers were analyzed by qPCR, normalized to host *CCR5,* and then fold change relative to mock-treated samples harvested 72 hrs post induction was determined. The change in viral transcripts normalized to host *TATA-box binding protein* (*TBP*) relative to mock-treated cells harvested at 72 h post induction was determined for putative latent genes *C1* and *ORF39* **(B)**, immediate-early lytic genes *ORF42* and *ORF43* **(C)**, the early lytic gene *ORF5* (**D**) and late lytic genes *ORF59* and *ORF45* **(E)** by qRT-PCR at the indicated times post-treatment. Treatment led to an increase of CalHV-3 transcripts, with a higher induction of lytic gene homologs compared to homologs of EBV latent genes. **(A)** – one-way ANOVA; **(B-E)** – two-way ANOVA. (** p < 0.01, ***p < 0.001, ****p < 0.0001). Fig 6A Created in BioRender. Krug, L. (2026) https://BioRender.com/fisf695.

Treatment of CJ0149 cells with 5 mM NaB in combination with 20 ng/ml of TPA led to an increase of all CalHV-3 transcripts examined over the 144 hours (hrs) time course, with higher induction of lytic gene homologs (*ORF42, ORF43, ORF5, ORF59, ORF45*) compared to homologs of EBV genes considered to be in the latent class (*C1, ORF39*) (Fig 6B–6E). The putative CalHV-3 *C1* latent gene, a homolog of EBV *latent membrane protein 1* (*LMP1)*, was induced 2.6-fold compared to uninduced cells at 144 hrs, and *ORF39*, the homolog of latent *EBV nuclear antigen 1* (*EBNA1*), increased by 3.5-fold (Fig 6B, blue bars). *ORF42* and *ORF43*, the respective homologs of the EBV lytic gene transactivators *BRLF1* and *BZLF1*, were induced by over 67-fold and 257-fold-(Fig 6C, green bars). The *gp07/ORF5* homolog of the putative early gene EBV viral DNA polymerase *BALF5* increased by 80-fold at 144 hrs (Fig 6D, purple bars). Lastly, genes of the late lytic class, *ORF59*, the homolog of the EBV small capsid protein *BFRF3*, and *ORF45*, the homolog of the EBV glycoprotein *gp350*, were induced by 340 and 820-fold, respectively, at 144 hrs (Fig 6E). Upon combinatorial treatment with TPA and NaB to induce reactivation (Fig 6A), the CalHV-3 genome copy number only increased 2.4-fold by qPCR. These observations indicate that CalHV-3 genes are transcribed in a pattern broadly consistent with latent and lytic gene expression programs of EBV homologs. In addition, quantitation of viral gene expression is a better indication of reactivation than viral DNA copy number in the CJ0149 cells.

### CalHV-3 genes were expressed in marmoset B-cell lymphomas

**Fig 7:**
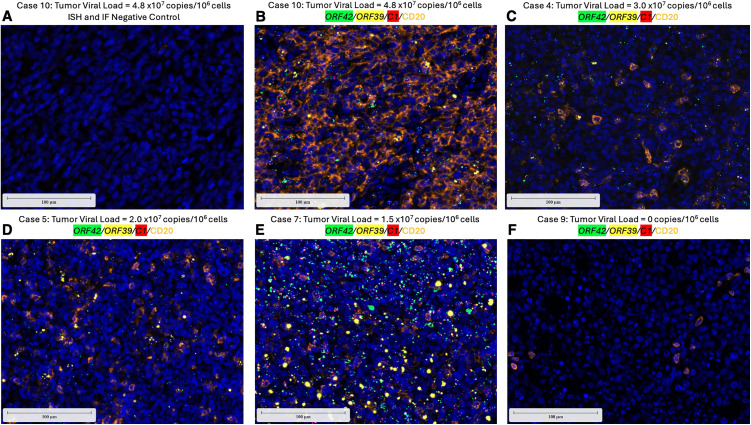
CalHV-3 viral transcripts were expressed in CalHV-3 associated B-cell lymphomas. Probes targeting CalHV-3 viral transcript were designed and applied to histopathologically-confirmed B-cell lymphomas from the common marmoset. **(A)** RNAscope ISH and IF negative control. Patterns of viral gene expression observed in neoplasms included the presence of all analyzed CalHV-3 transcripts (*ORF39, ORF42, C1*) in neoplastic cells, with *ORF42* being the highest expressed gene **(B-C)**. Other patterns included *ORF39* dominant expression with minimal *C1* **(D)** and regional *ORF42* and *ORF39* differential expression and high C1 expression throughout the tumor **(E)**. CalHV-3 viral transcripts were not detected in the histiocytic sarcoma **(F)**. OPAL 520, 570, 620, and 690 and DAPI fluorescence.

The expression of CalHV-3 viral transcripts representative of latent (*ORF39* and *C1*) and lytic (*ORF42*) genes classes was next analyzed in a subset of neoplasms by in situ hybridization (ISH; RNAscope, ACDBio), in combination with CD20 immunofluorescence (IF). CalHV-3 viral transcripts were identified within neoplastic CD20-positive B-cells in all analyzed cases of B-cell lymphoma (Fig 7A–7E), with multiple different patterns of observed viral gene expression noted in the examined neoplasms. Some lymphoma cases showed expression of all transcripts within the neoplastic population, with the highest expression of *ORF42*, followed by *ORF39* and then *C1* (Fig 7B and 7C). A case of B-cell lymphoma had higher expression of *ORF39* compared to *ORF42* in the neoplastic B-cells, with minimal observed *C1* expression (Fig 7D). One case displayed differential expression of CalHV-3 *ORF39* and *ORF42* in various regions of the lymphoma, with high *C1* expression throughout all regions (Fig 7E). CalHV-3 transcripts were not identified in the case of histiocytic sarcoma (Fig 7F), which was also negative for CalHV-3 DNA on ddPCR. Taken together, active CalHV-3 gene transcription within the B-cells of the tumor sections is additional support for the role of the virus in lymphomagenesis [26].

## Discussion

EBV has been associated with many diseases, including infectious mononucleosis, numerous malignancies, and neurodegenerative diseases such as MS [9]. Despite its etiological role in these diseases, effective preventatives and treatments that target EBV remain limited [6,7]. While animal models are essential for studying viruses and associated disorders, the exquisite host specificity of EBV for humans presents a challenge to study viral pathogenesis and therapeutic interventions [9,10]. By contrast, the infection of nonhuman primates with an endogenous gammaherpesvirus can serve as a preclinical model [9]. While CalHV-3 in the common marmoset was identified as a phylogenetically related gammaherpesvirus to EBV, biological similarities to EBV have not been well-described [13,15]. We characterized natural CalHV-3 infection in the common marmoset to validate its potential utility as a translational model of EBV. Our work shows that CalHV-3 has many biological similarities to human EBV infection, including persistent infection, viral shedding in saliva, and B-cell tropism. Additionally, CalHV-3 genes are generally transcribed in a pattern consistent with the latent and lytic expression programs of EBV homologs. Extremely high CalHV-3 viral loads are associated with all cases of B-cell lymphoma in the marmoset, and viral gene transcripts are expressed in the neoplastic tissue, suggesting a role for this herpesvirus in lymphomagenesis. These results indicate that CalHV-3 in the marmoset is an appropriate translational model system that can be utilized to further investigate the role of EBV in a multitude of disease processes, including neoplasia.

EBV is a ubiquitous herpesvirus in humans, with estimates of over 90% of the world’s adult population being infected [27]. In this study, the prevalence of CalHV-3 based on ddPCR positivity ranged from 19-31% in large-scale marmoset colony screenings, with the screening of other colonies with smaller sample sizes showing a prevalence of up to 63%. Previous reports of CalHV-3 prevalence estimated that roughly 60% of colonies were positive for CalHV-3 using alternative molecular methods, such as nested PCR [14]. The results of this study suggest that while all biomedical research colonies of common marmosets are likely positive for CalHV-3, the prevalence in colonies may vary. These prevalence rates may be influenced by a variety of factors, including husbandry practices, the housing environment, disease comorbidities, and breeding [28]. Future studies aim to further explore aspects that may contribute to CalHV-3 prevalence variability between colonies, including social housing and genetics, among others. Based on the large-scale screenings of three marmoset colonies, the prevalence of CalHV-3 increases with age, like EBV [29,30]. As interest in the marmoset as a model of aging continues to grow [31,32], CalHV-3 may be an important cofactor in these studies.

Serological testing is commonly utilized for EBV; however, the measurement of circulating viral load by molecular assays such as PCR may be a better representation of real-time infection status [30]. In studies of varying geographical regions and patient cohorts investigating PCR detection of EBV in healthy individuals, the prevalence of EBV DNA varies greatly but is generally lower than seroprevalence [30]. In a previous study, 79% (22/28) of tested marmosets had antibodies to CalHV-3 using an assay developed from an infected immortalized cell line [22]. This suggests that like EBV, CalHV-3 seroprevalence may be higher than the prevalence detected by molecular methods, such as the ddPCR assay used in this study. Unfortunately, no commercial serological assay is currently available for CalHV-3.

EBV infection is mainly transmitted in the saliva, with individual patients showing large variability in salivary viral loads over time [33]. Similarly, CalHV-3 is shed in the saliva of infected animals at viral loads that fluctuate dramatically over time. In both EBV and CalHV-3, saliva viral loads do not correlate with viral loads in PBMCs or blood [34]. While saliva ddPCR for CalHV-3 had a specificity of 100% and was able to identify all uninfected animal correctly, sensitivity was only 78% when compared to PBMC ddPCR at the same timepoint, with saliva not detecting CalHV-3 in some infected individuals [35]. Therefore, PBMC testing is likely necessary to most accurately identify animals infected with CalHV-3. Future studies aim to expand the scope of saliva sample collection to allow for more detailed longitudinal analyses and investigation of temporal or seasonal factors that may affect CalHV-3 shedding levels in infected animals.

The initial targets of EBV infection are B-cells, with life-long infection established in the memory B-cell population [36]. CalHV-3 similarly establishes lifelong infection in marmosets, with CalHV-3 remaining detectable in the PBMCs of infected animals over time. CD20 enriched B-cell populations had higher viral loads than PBMCs and B-cell depleted populations. This result is consistent with the known B-cell tropism of EBV [37]. The detection of EBV positive human subjects is enhanced by enriching CD19-positive B-cells from PBMCs [37]. In the marmosets of this study, CalHV-3 remained undetected in the enriched CD20-B-cell population of all four animals that were negative for CalHV-3 in PBMCs. While humans have roughly 5–10% of CD19-positive B-cells in PBMCs, marmoset have on average 25% CD20-positive B-cells [38,39], which may account for the increased sensitivity of ddPCR for CalHV-3 in marmoset PBMCs compared to EBV in human PBMCs. Additionally, in healthy humans, viral loads for EBV are low, with reports estimating 1–50 copies of EBV per million leukocytes [30,40]. In this study, CalHV-3 viral loads in asymptomatic animals are higher than those seen in humans asymptomatically infected with EBV, where thousands of copies of virus per million PBMCs are detectable in most infected marmosets. These higher circulating viral loads may be attributed in part to this larger population of circulating B-cells in marmosets compared to humans [38,39].

While neoplasia is generally uncommon in the marmoset compared to other nonhuman primates [41], lymphoma has been reported to be the most common neoplasm in this species [42]. The results of this study are consistent with these reports, with neoplasia present in only 1.8% (15 cases) of the 834 marmoset necropsies over a thirty-year period and lymphoma being suspected in most of those cases based on histologic morphology. Nine cases of B-cell lymphoma were confirmed by IHC in this study, including two cases of T-cell-rich large B-cell lymphoma (TCRLBCL), a subtype of B-cell lymphoma that has not been previously described in the common marmoset, but has been described in other nonhuman primate species in relationship to a lymphocryptovirus [43]. CalHV-3 was originally identified in association with cases of lymphoproliferation in the marmoset [13,15]. This study further strengthens an etiological association since all marmosets with B-cell lymphoma had extremely high viral loads (tens of millions of copies) in neoplastic tissue that were significantly higher than viral loads in other non-lymphoma tumors and nonneoplastic tissue (thousands of copies). While other tumors with corresponding normal nonneoplastic tissue were occasionally positive for CalHV-3, there was no significant difference in viral loads between these tissues (Fig 4C), providing no evident support for a role in the pathogenesis of these other neoplasms [44].

The extremely high viral loads (tens of millions of copies) of CalHV-3 in B-cell neoplasms suggests that CalHV-3 plays a causative and oncogenic role in a subtype of lymphomas in the common marmoset, similar to the role for EBV in humans [45,46]. While the specific mechanisms of EBV-driven oncogenesis remains unclear, EBV encodes many factors that interact with oncogenes and perturb the regulation of cell cycle and checkpoints that promote the development of B-cell neoplasms including Burkitt lymphoma and non-Hodgkin lymphoma [47,48]. For CalHV-3, homologues to EBV, including *ORF39* with homology to *EBNA1* and *C1* with homology to *LMP1*, have been identified [14,15]. Reactivation of CalHV-3 from an infected marmoset lymphoma-derived cell line upon treatment with two well-described agents broadly mirrored patterns of EBV gene expression, including the higher-fold increase in the expression of gene homologs classified as lytic compared to gene homologs classified as latent [49–51]. Multiple viral gene expression programs have been identified in EBV-associated B-cell lymphomas [48], and future studies of the CalHV-3 transcriptome will profile genome-wide expression patterns in marmoset B-cell lymphomas. CalHV-3-associated B-cell lymphoma in the common marmoset may be further developed as an important preclinical model to map the molecular mechanisms of viral-induced oncogenesis and to develop targeted therapeutics [52].

While large-scale prevalence screening was implemented in three biomedical research marmoset colonies, limitations on sample volume, frequency of sample collections, and availability of appropriate samples resulted in comparatively smaller sample sizes for some described analyses. Future studies aim to extend these analyses to additional samples, animals, and colonies in order to widen the analyses and results presented in this study.

In summary, this work expands our knowledge of CalHV-3 infection in the common marmoset to establish strong parallels with EBV infection in humans. Key features include persistent infection, fluctuations in salivary viral loads, B-cell tropism, profiles of latent and lytic viral gene expression, and oncogenesis in a small subset of infected animals. These findings establish CalHV-3 in the common marmoset as a clinically relevant NHP model to define determinants of EBV pathogenesis in cancer and other diseases and to aid in the development of anti-viral therapeutics and preventatives.

## Materials and methods

### Animal ethics, housing, and demographics

All work involving marmosets was included in active protocols reviewed and approved by the National Institute of Neurological Disorders and Stroke (NINDS), National Institute of Mental Health (NIMH), and University of Pittsburgh (Pitt) Animal Care and Use Committees (ACUC). Animals were housed at AAALAC-accredited facilities following USDA regulations and the Guide for the Care and Use of Laboratory Animals by the United States National Research Council. Demographic information for animals, including sex and date of birth, was obtained from colony records. For this study, animals were classified into the following age groups based on previously described characteristics of phenotypic aging in the common marmoset: young (less than 24 months/2 years), adult (24–59 months/2–5 years), aging (60–95 months/5–8 years), and aged (96 months/8 years and older) [31,53]. Age at sample collection was rounded to the nearest month.

### Sedation and anesthesia

Animals were sedated for sample collection procedures. Sedation was achieved by intramuscular injection of ketamine (10 mg/kg) and dexmedetomidine (4 mg/kg) or isoflurane inhalation by facemask (2–3%). Injectable anesthesia was reversed with an intramuscular injection of atipamezole.

### Blood and saliva collection

Whole blood was collected for routine health surveillance and approved experimental procedures. Peripheral blood mononuclear cells (PBMCs) were isolated from the whole blood using lymphocyte separation medium. The PBMCs were collected and washed with phosphate-buffered saline (PBS). PBMCs were pelleted and stored at -80°C for DNA extraction. Cell separation with or without flow cytometry was also performed on fresh PBMCs in a subset of samples.

Saliva was collected on sterile cotton swabs and isolated by spinning the swab in a microcentrifuge tube at 1500rpm for 15 minutes. It was then stored at -20°C until DNA extraction. In a subset of animals from the NINDS colony, PBMCs and saliva were both collected at multiple time points.

### Cell culture and chemical treatment

The marmoset B lymphoblastoid cell line infected with CalHV-3 (CJ0149) was cultured in RPMI 1640 supplemented with 10% fetal bovine serum, 100 U/mL of penicillin, 100 μg/mL of streptomycin (Corning, Tewksbury, MA), and 2 mM L-glutamine (Corning) at 37°C in 5% CO_2_. 10x10^6^ of CJ0149 cells at 0.3x10^6^/ml were treated with the combination of 20 ng/mL 2-O-Tetradecanoylphorbol-13-acetate (TPA) (Millipore Sigma, St Louis, MO) and 5 mM sodium butyrate (NaB) (Millipore Sigma).

### DNA and RNA extraction

DNA was extracted from PBMCs and untreated or chemically induced CJ0149 cells using the DNeasy Blood & Tissue Kit (Qiagen Sciences, Germantown, MD) according to manufacturer specifications. DNA was extracted from saliva using the QIAamp MinElute Virus Spin Kit (Qiagen) per manufacturer protocol. DNA was extracted from formalin-fixed paraffin-embedded (FFPE) tissue using the QIAamp DNA FFPE Advanced Kit (Qiagen) according to the manufacturer’s instructions. DNA concentrations and 260/280 ratios were measured using a Nanodrop 2000 UV-Vis spectrophotometer.

RNA was extracted from untreated and chemically induced CJ0149 cells with RNeasy Plus kit (Qiagen) and then digested with DNase (Thermo Fisher Scientific, Waltham, MA) at 37°C.

### Quantitative PCR (qPCR)

40 ng of DNA was amplified for qPCR with PowerUP SYBR Green Master Mix (Thermo Fisher Scientific) using 0.5 µM of primers specific for the CalHV-3 *gp07* viral polymerase gene [14,17] and the marmoset *C-C motif chemokine receptor 5* (*CCR5)* gene as the endogenous control (S1 Table). Relative levels of CalHV-3 DNA were measured using delta-delta cycle threshold (Ct) method compared to uninduced control conditions after normalizing to the marmoset *CCR5* gene locus.

### Droplet digital PCR (ddPCR)

The general droplet digital (ddPCR) procedure was performed as previously described [54]. Briefly, primers were used to amplify the CalHV-3 *gp07* viral DNA polymerase gene and the marmoset housekeeping gene *beta-actin* (S1 Table) [55]. Probes were fluorescently labeled, with a probe for CalHV-3 *gp07* FAM-MGBNFQ-labeled, while *beta-actin* probes were VIC-MGBNFQ-labeled (S1 Table). For each DNA sample, primers and probes for CalHV-3 were duplexed with beta-actin, with the final concentrations of 900 nM per primer and 250 nM per probe.

Each sample was analyzed using duplicate wells and the QuantaSoft software, version 1.7.4.0917 (Bio-Rad, Hercules, CA). Droplet positivity was determined by fluorescence intensity, with droplets considered positive when they were above the manually determined threshold. Target copies per μL were calculated by averaging replicate wells for each sample. Cellular DNA was calculated by halving the number of *beta-actin* copies to account for two copies of the housekeeping gene *beta-actin* per cell. Results are reported as copies of virus per 10^6^ cells (PBMCs and FFPE tissue) or copies of virus per mL (saliva). PBMC viral loads were considered low (less than 10,000 copies of CalHV-3/10^6^ PBMCs), moderate (10,000–100,000 copies of CalHV-3/10^6^ PBMCs), high (100,001–1,000,000 copies/10^6^ PBMCs), and extremely high (more than 1,000,000 copies/10^6^ PBMCs).

### Quantitative-reverse transcription PCR (q-RT-PCR)

500 ng of DNase-treated RNA extracted from the CJ0149 cell line was reverse transcribed with random hexamer and SuperScript IV reverse transcriptase (Thermo Fisher Scientific). Quantitative real-time PCR was conducted on QuantStudio 3 Real-Time PCR machine (Thermo Fisher Scientific) using PowerUp SYBR Green Master Mix (Thermo Fisher Scientific) and gene-specific primers in S1 Table. The relative amount of transcript from genes of interest was measured through the delta-delta Ct method by comparing to control conditions after normalizing to the marmoset *TATA-box binding protein* (*TBP)* housekeeping gene [56].

### Cell separation

Manual column-based magnetic cell separation and B-cell positive selection was performed in a subset of PBMC samples from the NINDS and NIMH colonies. PBMCs were labeled with a mouse anti-human CD20-Pacific Blue antibody (clone B-Ly1; SouthernBiotech, Birmingham, AL). Anti-mouse IgG microbeads (Miltenyi Biotec, Auburn, CA) were added, and labeled B-cells were magnetically separated by passage through MS columns (Miltenyi Biotec, Auburn, CA). From these samples, ddPCR was performed on unmanipulated PBMCs, the positively selected B-cell enriched population, and the negatively selected B-cell depleted population.

### Flow cytometry

In a subset of fresh PBMCs with a positively selected B-cell enriched population and a negatively selected B-cell depleted population, flow cytometry was performed to characterize the populations. In addition to the previously mentioned anti-human CD20-Pacific Blue antibody, extracellular antibodies included Ghost Dye Violet 540 (Cytek Biosciences, Fremont CA), anti-marmoset CD45-PE (BioLegend, San Diego, CA), anti-human CD3-BUV395 (clone SP34–2; BD Biosciences, Franklin Lakes, NJ), anti-human CD4-BUV805 (clone L200; BD Biosciences, Franklin Lakes, NJ), anti-human CD8-APC (clone LT8; Thermo Fisher Scientific), anti-human CD56-PE-Cyanine7 (clone MY31; Cytek Biosciences, Fremont CA), and anti-human CD14-PerCP-Cy5.5 (clone M5E2; BD Biosciences, Franklin Lakes, NJ). Samples were run in 96-well plates using a Cytek Aurora. Data analysis was performed using FlowJo version 10.9.0.

### Euthanasia

Euthanasia was performed as described in the approved protocol or as directed by the veterinarian due to clinical disease. Methods of euthanasia included transcardial perfusion of anesthetized animals with PBS or fixative and intravenous administration of pentobarbital to anesthetized animals, as approved in animal study protocols. Collected tissues were fixed in 10% neutral buffered formalin for approximately a week prior to paraffin embedding.

### Tissue archive query

The Division of Veterinary Resources (DVR) database at the National Institutes of Health was searched for marmoset necropsies from 1992 to 2022. The necropsies were then queried for the diagnosis of neoplasia. Archived specimens, including hematoxylin and eosin (H&E) slides and FFPE tissue blocks, for nine cases of suspected lymphoma and five cases of other neoplasms were retrieved and reviewed.

### Histopathology and immunohistochemistry (IHC)

Formalin-fixed tissues were trimmed into sections, and sections were embedded into paraffin (Histoserv, Germantown, MD). Paraffin blocks were sectioned at 5 μm with a manual microtome and were mounted on positively charged glass slides. Sections were stained routinely with H&E. IHC was also performed using a BOND RX^m^ and the BOND Poly^m^er Refine Detection kit (Leica Biosystems, Deer Park, IL). In suspected cases of lymphoma, IHC was performed on tumor tissue using polyclonal rabbit anti-human CD3 (1:400; EDTA pH 9 heat-induced epitope retrieval; Agilent, Santa Clara, CA) and monoclonal mouse anti-human CD20 (Clone L26; 1:1600; citrate pH 6 heat-induced epitope retrieval; Agilent, Santa Clara, CA) to further classify the neoplasm. If neoplastic tissue was negative for CD3 and CD20, IHC was performed using a polyclonal rabbit anti-human Iba1 (1:1600; citrate pH 6 heat-induced epitope retrieval; FUJIFILM Wako Chemicals U.S.A. Corporation) to determine if the neoplasm was histiocytic in origin. Slides were optically scanned as a whole slide digital image using an AT2 digital slide scanner (Leica Biosystems, Deer Park, IL) at 20x (0.5 μm per pixel). Slide images were reviewed using HALO Link (Indica Labs, Albuquerque, NM) via web browser.

### RNAscope

The expression of CalHV-3 genes was detected in 5 μm FFPE marmoset tissue sections with the RNAScope 2.5 LS V probes CalHV3-ORF42-C1 spanning 72,699–74,525 nt of NC_004367.1 (Advanced Cell Diagnostics (ACD), Newark, CA), CalHV3-ORF39-C2 spanning the reverse complement of 70,133–69,150 nt (ACD), and CalHV3-C1-O1-C3 spanning the exons 484–721, 811–897 and 1087–1829 nt of C1 (ACD) with the RNAscope LS Multiplex Fluorescent Assay (ACD) using the Bond RX auto-stainer (Leica Biosystems) with a tissue pretreatment of 30 min at 100°C with EDTA pH9 and 1:750 dilution of OPAL 520, OPAL 570, or OPAL 690 reagents (Akoya Biosciences, Marlborough, MA), respectively. Immunofluorescence for B-cells was performed after RNAScope using mouse anti-human CD20, clone L26 (Agilent) at a 1:200 dilution for 30 min using the Bond Polymer Refine Kit (Leica Biosystems) minus DAB and Hematoxylin with 1:750 dilution of OPAL 620 reagent for 30 min. The RNAscope 3-plex LS Multiplex Negative Control Probe [Bacillus subtilis dihydrodipicolinate reductase (dapB) gene in channels C1, C2, and C3] followed by IHC with no primary antibody was used as an ISH and IHC negative control. Embedded pellets of CJ0149 cells were used as the positive control for the initial optimization of the CalHV-3 gene probes. Slides were digitally imaged using an PhenoImager HT 2.0 (Akoya Biosciences). Slide images were reviewed using HALO Link (Indica Labs) via web browser. Snapshots of slide images were downloaded directly from HALO Link.

### Statistical analysis

The prevalence of CalHV-3 in colonies and age groups was calculated. Fisher’s exact tests were performed to determine the relationship between age, sex, and CalHV-3 status. One-way ANOVA analyses or unpaired t-tests were performed to determine differences in the means of the CalHV-3 viral loads between colonies, age groups, and sexes. A contingency table was constructed, and the sensitivity, specificity, and positive and negative predictive values were calculated; a Fisher’s exact test was performed for the detection of CalHV-3 infection in saliva versus PBMCs. The Pearson r coefficient was calculated between saliva and PBMC viral loads. A one-way ANOVA analysis and unpaired or paired t-tests were performed to determine differences in mean viral loads between lymphoma, other neoplasms, and non-neoplastic tissue. One- or two-way ANOVA analyses determined significant differences between fold changes in gene expression. These statistical analyses were performed using GraphPad Prism Version 9.3.1 (GraphPad Software, San Diego, CA).

## Supporting information

S1 Fig CalHV-3 is genetically related to EBV. Features of the CalHV-3 genome (NC_004367), including the protein coding sequence (CDS), variations such as genome deletions, and repeated regions, are mapped to the positive and negative strands of the EBV genome (NC_007605.1), highlighting positional homologues to EBV genes. Basic Local Alignment Search Tool (BLAST) results highlight regions of similarities between the genomes on the inner ring. The CalHV-3 genome features and BLAST search were generated using Proksee, a web tool for characterization and visualization of genomes [16].
(PDF)

S2 Fig Males and females were equally infected with CalHV-3. (A) The male to female sex ratio was approximately 1 in each colony. (B) Within each colony, CalHV-3 infected animals included roughly the same proportion of males and females.
(PDF)

S3 Fig Contemporaneous saliva and PBMC samples from 49 animals were utilized to determine the association between CalHV-3 detection in PBMCs and saliva. (PDF)

S4 Fig Flow cytometry characterized marmoset PBMCs and magnetically separated B-cell enriched and B-cell depleted populations. A flow cytometry panel with optimized for PBMCs (A), the negatively selected B-cell depleted population (B), and the positively selected B-cell enriched population (C). Representative gating strategy shown is from Animal J. (D) Percentage of CD20-positive B-cells in PBMCs, the negatively selected B-cell depleted population, and the positively selected B-cell enriched population in the four samples with flow cytometry analysis.
(PDF)

S5 Fig Fifteen cases of marmoset neoplasia were identified and analyzed in this study. (PDF)

S6 Fig Non-lymphoma neoplasms were rarely diagnosed in the common marmoset. The intestinal adenocarcinomas in case 11 and 12 were characterized by atypical villous and gland formations with mucin producing cells (A and B). In case 13 (C), a perianal sarcoma is characterized by undifferentiated spindle cells. The mucinous adenocarcinoma in case 15 was present in the mesentery, with anaplastic mucinous cells of unknown origin (D). In case 14 (E), a parathyroid carcinoma invades through its capsule and into the surrounding thyroid gland. H&E.
(PDF)

S1 Table Primers and probes for CalHV-3 and marmoset genes were used in qPCR, q-RT-PCR, and ddPCR. * 3’ Probe Quencher used was MGBNFQ. F = forward, R = reverse, CJ = *Callithrix jacchus.*
(PDF)

S2 Table Fifteen cases of neoplasia in the common marmoset were analyzed in this study. NA = not available; TCRLBCL = T-cell rich large B-cell lymphoma.
(PDF)

S3 Table Extremely high levels of CalHV-3 DNA were detected in all cases of lymphoma in the common marmoset. NA = not available; TCRLBCL = T-cell rich large B-cell lymphoma.
(PDF)

S1 Data Data generated in this study and utilized for this manuscript and figures. (XLSX)
