# Supplementary material for: Callitrichine herpesvirus 3 in the common marmoset is a model of Epstein-Barr virus infection and associated lymphoma
Source: PLoS Pathog. 2026 Jul 17;22(7):e1014450. doi: 10.1371/journal.ppat.1014450 (PMC13395367; doi:10.1371/journal.ppat.1014450)
Supplement: S1 Fig — Features of the CalHV-3 genome (NC_004367), including the protein coding sequence (CDS), variations such as genome deletions, and repeated regions, are mapped to the positive and negative strands of the EBV genome (NC_007605.1), highlighting positional homologues to EBV genes. Basic Local Alignment Search Tool (BLAST) results highlight regions of similarities between the genomes on the inner ring. The CalHV-3 genome features and BLAST search were generated using Proksee, a web tool for characterization and visualization of genomes [16]. (PDF) [file ppat.1014450.s001.pdf]

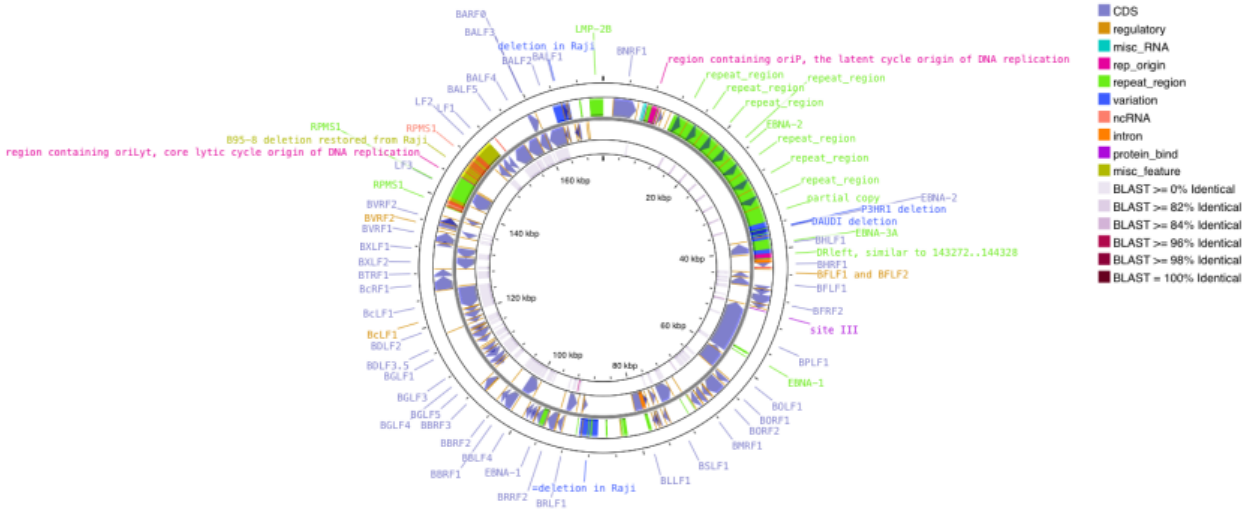

S1 Fig. CalHV-3 is genetically related to EBV. Features of the CalHV-3 genome (NC_004367), including the protein coding sequence (CDS), variations such as genome deletions, and repeated regions, are mapped to the positive and negative strands of the EBV genome (NC_007605.1), highlighting positional homologues to EBV genes. Basic Local Alignment Search Tool (BLAST) results highlight regions of similarities between the genomes on the inner ring. The CalHV-3 genome features and BLAST search were generated using Proksee, a web tool for characterization and visualization of genomes (16).
