# Supplementary material for: Callitrichine herpesvirus 3 in the common marmoset is a model of Epstein-Barr virus infection and associated lymphoma
Source: PLoS Pathog. 2026 Jul 17;22(7):e1014450. doi: 10.1371/journal.ppat.1014450 (PMC13395367; doi:10.1371/journal.ppat.1014450)
Supplement: S2 Fig — (A) The male to female sex ratio was approximately 1 in each colony. (B) Within each colony, CalHV-3 infected animals included roughly the same proportion of males and females. (PDF) [file ppat.1014450.s002.pdf]

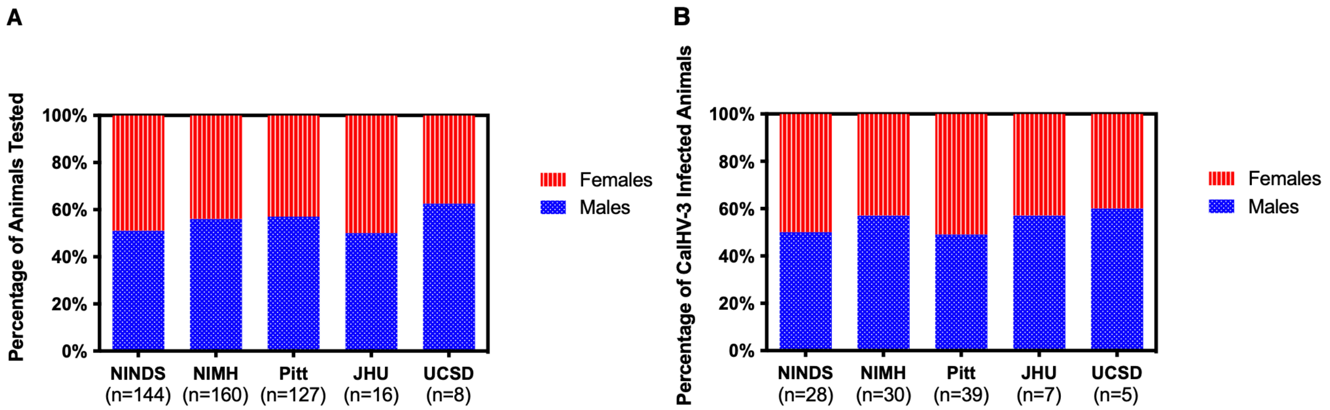

S2 Fig. Males and females were equally infected with CalHV-3. (**A**) The male to female sex ratio was approximately 1 in each colony. (**B**) Within each colony, CalHV-3 infected animals included roughly the same proportion of males and females.
