# Supplementary material for: Callitrichine herpesvirus 3 in the common marmoset is a model of Epstein-Barr virus infection and associated lymphoma
Source: PLoS Pathog. 2026 Jul 17;22(7):e1014450. doi: 10.1371/journal.ppat.1014450 (PMC13395367; doi:10.1371/journal.ppat.1014450)
Supplement: S3 Fig — (PDF) [file ppat.1014450.s003.pdf]

PBMC CalHV-3 Status	Saliva CalHV-3 Status
Positive n = 18	78% (14/18) positive
	22% (4/18) negative
Negative n = 31	0% (0/31) positive
	100% (31/31) negative

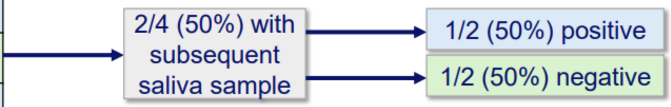

S3 Fig. Contemporaneous saliva and PBMC samples from 49 animals were utilized to determine the association between CalHV-3 detection in PBMCs and saliva.
