# Supplementary material for: Callitrichine herpesvirus 3 in the common marmoset is a model of Epstein-Barr virus infection and associated lymphoma
Source: PLoS Pathog. 2026 Jul 17;22(7):e1014450. doi: 10.1371/journal.ppat.1014450 (PMC13395367; doi:10.1371/journal.ppat.1014450)
Supplement: S4 Fig — A flow cytometry panel with optimized for PBMCs (A), the negatively selected B-cell depleted population (B), and the positively selected B-cell enriched population (C). Representative gating strategy shown is from Animal J. (D) Percentage of CD20-positive B-cells in PBMCs, the negatively selected B-cell depleted population, and the positively selected B-cell enriched population in the four samples with flow cytometry analysis. (PDF) [file ppat.1014450.s004.pdf]

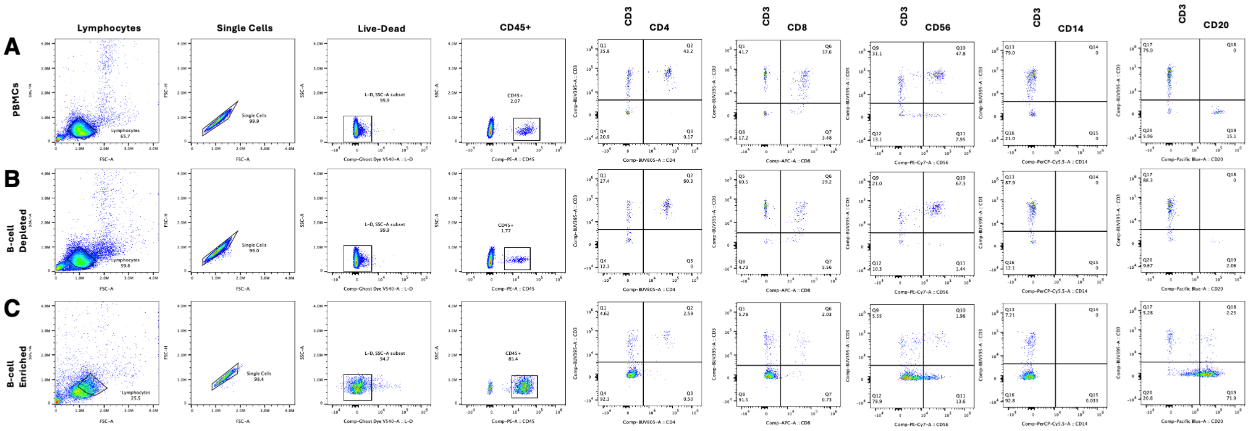

D

Animal	Percentage of B-cells in PBMCs	Percentage of B-cells in B-cell depleted population	Percentage of B-cells in B-cell enriched population
G	38.8	2.52	88.6
H	20.4	0.65	86.5
I	9.46	0	85.2
J	15.1	2.06	71.9

S4 Fig. Flow cytometry characterized marmoset PBMCs and magnetically separated B-cell enriched and B-cell depleted populations. A flow cytometry panel with optimized for PBMCs (A), the negatively selected B-cell depleted population (B), and the positively selected B-cell enriched population (C). Representative gating strategy shown is from Animal J. (D) Percentage of CD20-positive B-cells in PBMCs, the negatively selected B-cell depleted population, and the positively selected B-cell enriched population in the four samples with flow cytometry analysis.
