# Supplementary material for: Callitrichine herpesvirus 3 in the common marmoset is a model of Epstein-Barr virus infection and associated lymphoma
Source: PLoS Pathog. 2026 Jul 17;22(7):e1014450. doi: 10.1371/journal.ppat.1014450 (PMC13395367; doi:10.1371/journal.ppat.1014450)
Supplement: S5 Fig — (PDF) [file ppat.1014450.s005.pdf]

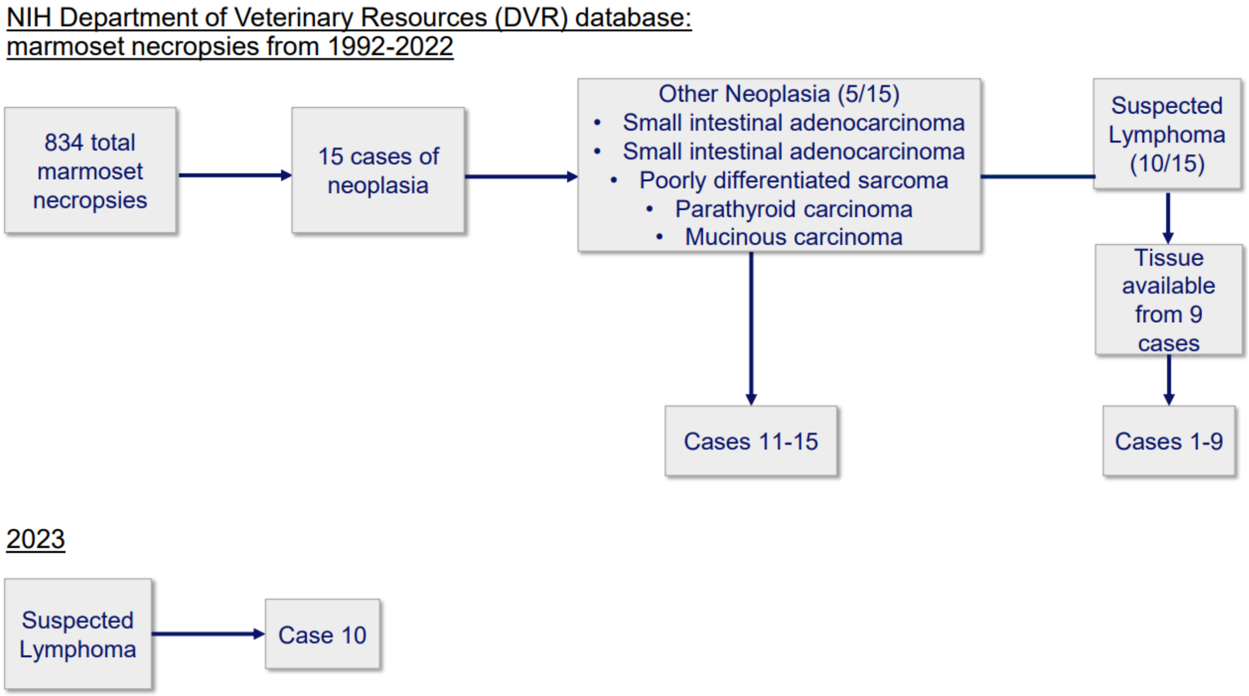

S5 Fig. Fifteen cases of marmoset neoplasia were identified and analyzed in this study.
