# Supplementary material for: Callitrichine herpesvirus 3 in the common marmoset is a model of Epstein-Barr virus infection and associated lymphoma
Source: PLoS Pathog. 2026 Jul 17;22(7):e1014450. doi: 10.1371/journal.ppat.1014450 (PMC13395367; doi:10.1371/journal.ppat.1014450)
Supplement: S6 Fig — The intestinal adenocarcinomas in case 11 and 12 were characterized by atypical villous and gland formations with mucin producing cells (A and B). In case 13 (C), a perianal sarcoma is characterized by undifferentiated spindle cells. The mucinous adenocarcinoma in case 15 was present in the mesentery, with anaplastic mucinous cells of unknown origin (D). In case 14 (E), a parathyroid carcinoma invades through its capsule and into the surrounding thyroid gland. H&E. (PDF) [file ppat.1014450.s006.pdf]

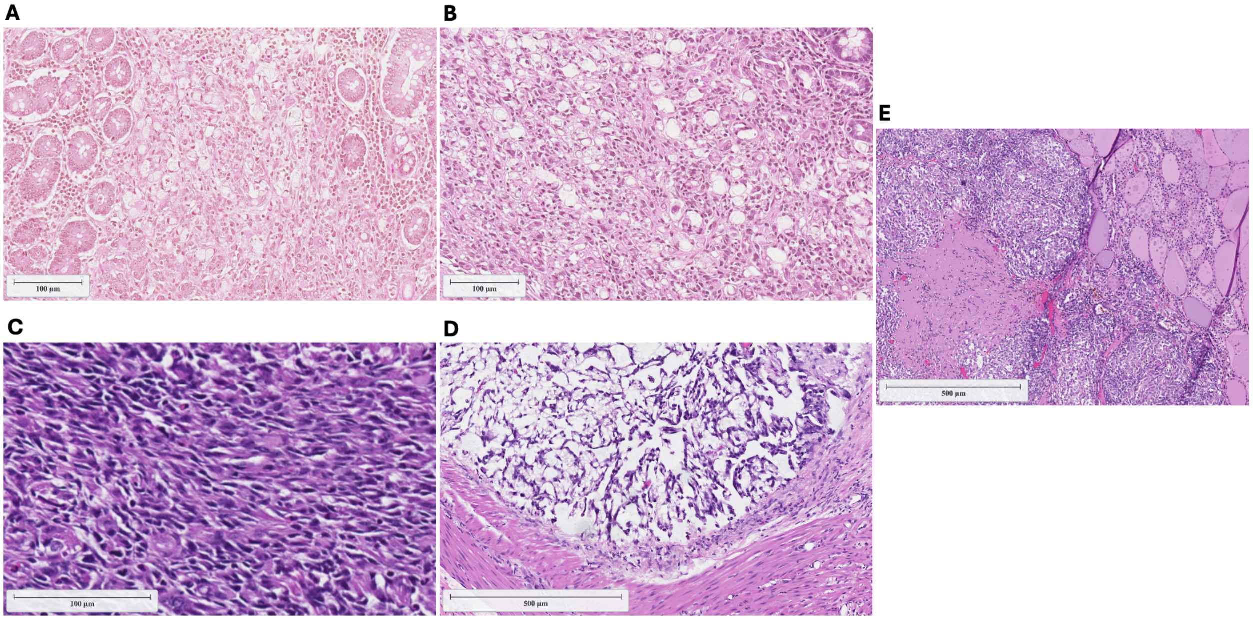


**S6 Fig. Non-lymphoma neoplasms were rarely diagnosed in the common marmoset.** The intestinal adenocarcinomas in case 11 and 12 were characterized by atypical villous and gland formations with mucin producing cells (A and B). In case 13 (C), a perianal sarcoma is characterized by undifferentiated spindle cells. The mucinous adenocarcinoma in case 15 was present in the mesentery, with anaplastic mucinous cells of unknown origin (D). In case 14 (E), a parathyroid carcinoma invades through its capsule and into the surrounding thyroid gland. H&E.
