# Supplementary material for: Callitrichine herpesvirus 3 in the common marmoset is a model of Epstein-Barr virus infection and associated lymphoma
Source: PLoS Pathog. 2026 Jul 17;22(7):e1014450. doi: 10.1371/journal.ppat.1014450 (PMC13395367; doi:10.1371/journal.ppat.1014450)
Supplement: S1 Table — * 3’ Probe Quencher used was MGBNFQ. F = forward, R = reverse, CJ = Callithrix jacchus. (PDF) [file ppat.1014450.s007.pdf]

Name	Sequence	Application
CalHV-3_*gp07*_F	AAACCGGGTTCCCATATCAG	q-RT-PCR, qPCR, ddPCR
CalHV-3_*gp07*_R	TGCCACAAACACGTATGGAA	q-RT-PCR, qPCR, ddPCR
CalHV-3_*gp07*_FAM	AAGCACGCAGATGCCCCA	ddPCR probe*
CalHV-3_*C1*_F	CGTTACTCTTACCCTTGCTTTCTAG	q-RT-PCR
CalHV-3_*C1*_R	CGAGAATGGCGATTAAGATCAAG	q-RT-PCR
CalHV-3_*ORF42*_F	GGTTTACCTAGGGCTTCATACG	q-RT-PCR
CalHV-3_*ORF42*_R	TGTTCGTCTACAGGTGTCATG	q-RT-PCR
CalHV-3_*ORF43*_F	GAAAACGATACAGAAACCGCC	q-RT-PCR
CalHV-3_*ORF43*_R	AAGGGTAGGACACATTTGACG	q-RT-PCR
CalHV-3_*ORF45*_F	GCCAATCTGTACCAAGCAATG	q-RT-PCR
CalHV-3_*ORF45*_R	TTCCCTCTGTGAAATGTCCTG	q-RT-PCR
CalHV-3_*ORF59*_F	AATGTGCAAAGTAGGCTAGAGG	q-RT-PCR
CalHV-3_*ORF59*_R	TTCGGCGATAAGGAATGTCAG	q-RT-PCR
CalHV-3_*ORF39*_F	ACTAAACCGCAGACATTCTCC	q-RT-PCR
CalHV-3_*ORF39*_R	AAAGAGGGCTGATGCGTAC	q-RT-PCR
CJ_*TBP*_F	CCATGACTCCCGGAATCCCTA T	q-RT-PCR
CJ_*TBP*_R	ATAGGCTGTGGGGTCAGTCCA	q-RT-PCR
CJ_*CCR5*_F	CAGCCCTCATTTTCCATTCAG	qPCR
CJ_*CCR5*_R	GATTCCCGAGTAGCAGATGAC	qPCR
CJ_*beta-actin*_F	AGGCGCACAGTAGGTCTGAA	ddPCR
CJ_*beta-actin*_R	TGCCACAAACACGTATGGAA	ddPCR
CJ_*beta-actin*_VIC	AAGCACGCAGATGCCCCA	ddPCR probe*

S1 Table. Primers and probes for CalHV-3 and marmoset genes were used in qPCR, q-RT-PCR, and ddPCR. * 3' Probe Quencher used was MGBNFQ. F = forward, R = reverse, CJ = *Callithrix jacchus*.
