# Supplementary material for: Callitrichine herpesvirus 3 in the common marmoset is a model of Epstein-Barr virus infection and associated lymphoma
Source: PLoS Pathog. 2026 Jul 17;22(7):e1014450. doi: 10.1371/journal.ppat.1014450 (PMC13395367; doi:10.1371/journal.ppat.1014450)
Supplement: S2 Table — NA = not available; TCRLBCL = T-cell rich large B-cell lymphoma. (PDF) [file ppat.1014450.s008.pdf]

Case Number	Diagnosis	Age (Years)	Sex	Clinical Findings	Gross Findings
1	TCRLBCL (multiple organs affected)	NA	M	Lethargy, abdominal mass	Enlarged spleen and mesenteric lymph nodes
2	B-cell lymphoma (gastrointestinal)	9	F	Vomiting, abdominal mass	Ileal mass
3	TCRLBCL (gastrointestinal)	NA	M	Lethargy, abdominal mass	Ileal mass, enlarged mesenteric lymph nodes
4	B-cell lymphoma (multiple organs affected)	10	M	None	Enlarged spleen and mesenteric lymph nodes
5	B-cell lymphoma (multiple organs affected)	10	M	Chronic weight loss and diarrhea	Enlarged mesenteric lymph nodes
6	B-cell lymphoma (multiple organs affected)	4	M	None	Enlarged mesenteric lymph nodes
7	B-cell lymphoma (gastrointestinal)	6	F	Chronic weight loss and diarrhea	Dilated gastrointestinal tract
8	B-cell lymphoma (gastrointestinal)	6	F	Chronic weight loss	None
9	Histiocytic sarcoma (gastrointestinal)	5	M	Abdominal distension	Ascites, enlarged mesenteric lymph nodes
10	B-cell lymphoma (multiple organs affected)	5	M	Chronic weight loss and diarrhea	Enlarged mesenteric and colonic lymph nodes
11	Small intestinal adenocarcinoma	4	F	Weight loss, decreased appetite	Ascites, small yellow-tan foci on mesenteric border of jejunum
12	Small intestinal adenocarcinoma	8	F	Abdominal distension and subcutaneous edema	Ascites, thickened duodenum with prominent lymphatics
13	Poorly differentiated sarcoma	1	M	Chronic rectal prolapse	Reddish to tan perianal mass
14	Parathyroid carcinoma	8	M	None	None
15	Mucinous adenocarcinoma	8	F	None	Small white nodules on surface of liver and abdominal muscle

S2 Table. Fifteen cases of neoplasia in the common marmoset were analyzed in this study. NA = not available; TCRLBCL = T-cell rich large B-cell lymphoma
