# Supplementary material for: Callitrichine herpesvirus 3 in the common marmoset is a model of Epstein-Barr virus infection and associated lymphoma
Source: PLoS Pathog. 2026 Jul 17;22(7):e1014450. doi: 10.1371/journal.ppat.1014450 (PMC13395367; doi:10.1371/journal.ppat.1014450)
Supplement: S3 Table — NA = not available; TCRLBCL = T-cell rich large B-cell lymphoma. (PDF) [file ppat.1014450.s009.pdf]

Case Number	Neoplasm Diagnosed	CalHV-3 Viral Load in Neoplastic Tissue (copies/10^6 cells)	CalHV-3 Viral Load in Non-Neoplastic Tissue (copies/10^6 cells)	CalHV-3 Viral Load in PBMCs (copies/10^6 PBMCs)
1	TCRLBCL	2.5×10^7	NA	NA
2	B-cell lymphoma	3.0×10^7	NA	NA
3	TCRLBCL	3.8×10^7	NA	NA
4	B-cell lymphoma	3.0×10^7	NA	NA
5	B-cell lymphoma	2.0×10^7	NA	NA
6	B-cell lymphoma	3.1×10^7	NA	NA
7	B-cell lymphoma	1.5×10^7	NA	1.3×10^6
8	B-cell lymphoma	3.1×10^6	NA	NA
9	Histiocytic sarcoma	0	0	0
10	B-cell lymphoma	4.8×10^7	NA	1.7×10^6
11	Small intestinal adenocarcinoma	4.3×10^4	8.0×10^3	NA
12	Small intestinal adenocarcinoma	8.0×10^5	2.5×10^4	NA
13	Poorly differentiated sarcoma	0	0	NA
14	Parathyroid carcinoma	2.1×10^3	5.2×10^2	NA
15	Mucinous adenocarcinoma	2.5×10^4	1.3×10^4	NA

S3 Table. Extremely high levels of CalHV-3 DNA were detected in all cases of lymphoma in the common marmoset. NA= not available; TCRLBCL = T-cell rich large B-cell lymphoma
